# Plasma Lipidomic Alterations in Fontan Circulation Reflect Cardiovascular Functional Reserve

**DOI:** 10.3390/metabo15090592

**Published:** 2025-09-07

**Authors:** Arun Surendran, Amir Ravandi, Ashish H. Shah

**Affiliations:** 1 Mass Spectrometry Core Facility, BRIC-Rajiv Gandhi Centre for Biotechnology (RGCB), Thiruvananthapuram, Kerala 695014, India; arunsurendran@rgcb.res.in; 2 Cardiovascular Lipidomics Laboratory, St. Boniface Hospital, Albrechtsen Research Centre, Winnipeg, MB R2H 2A6, Canada; amir.ravandi@umanitoba.ca; 3 Department of Physiology and Pathophysiology, Rady Faculty of Health Sciences, University of Manitoba, Winnipeg, MB R2H 2A6, Canada; 4 Section of Cardiology, Department of Internal Medicine, Rady Faculty of Health Sciences, University of Manitoba, Winnipeg, MB R2H 2A6, Canada; 5 Precision Cardiovascular Medicine Group, St. Boniface Hospital Research, Winnipeg, MB R2H 2A6, Canada

**Keywords:** Fontan circulation, lipidomics, LPC (lysophosphatidylcholine), LPC(O) [ether-linked LPC], PS (phosphatidylserines), cardiovascular physiology

## Abstract

**Background:** Fontan circulation is associated with impaired cardiac output, reduced exercise capacity, and systemic metabolic stress. However, the underlying lipidomic alterations remain poorly defined. **Methods:** Using targeted mass spectrometry, we analyzed 291 lipid species in fasting plasma samples from 20 adults with Fontan circulation and 20 age- and sex-matched healthy controls. **Results:** Forty-eight lipids were significantly altered between groups (*p* < 0.05), including reductions in total lysophosphatidylcholines (LPC) and total ether-linked LPC [LPC(O)] and elevations in total phosphatidylserines (PS). Notably, LPC(O-22:1) and LPC(O-20:0) were decreased, while PS 40:5 was elevated, with several of these species demonstrating strong correlations (|r| > 0.5, *p* < 0.001) with the stroke index, cardiac index, and VO_2_max. These three lipid species also showed excellent capability in discriminating Fontan patients from healthy controls (AUC > 0.78). Correlation network and pathway enrichment analyses revealed tightly coordinated lipid clusters containing LPC/LPC(O), PE, and PS species as central features of dysregulated Fontan metabolism. **Conclusions:** These exploratory findings highlight coordinated lipid alterations associated with impaired cardiovascular reserve in Fontan patients. While based on unadjusted *p*-values and therefore being hypothesis-generating, they provide novel insight into the metabolic landscape of Fontan physiology and warrant validation in larger, independent cohorts.

## 1. Introduction

Lipid metabolism plays a central role in cardiovascular physiology, regulating energy homeostasis, membrane composition, inflammatory signalling, and vascular function. In various cardiovascular conditions, including heart failure, atherosclerosis, and stroke, lipidomic remodelling has emerged both as a marker and a mediator of disease progression [1,2]. Fontan circulation, a unique surgical repair created by directly connecting the vena cavae to the pulmonary circulation, results in higher venous pressure and a low cardiac output phenotype [3]. Although this repair separates the systemic and pulmonary circulations, preserves systemic oxygen saturation, and prevents volume overload of the single functional ventricle, the resulting hemodynamic state ultimately leads to multisystem dysfunction as these patients reach adulthood [4].

Because there is no subpulmonary ventricle, pulmonary blood flow depends on passive venous return driven by elevated central venous pressure, which chronically exposes the liver and other organs to venous congestion [3,5,6]. This circulation is inherently inefficient, with limited preload to the systemic ventricle and an inability to augment cardiac output during exercise [7,8]. Over time, these physiological constraints contribute to complications such as Fontan-associated liver disease, protein-losing enteropathy, renal dysfunction, and reduced exercise tolerance [9,10,11]. Thus, while life-saving, the Fontan circulation represents a fragile equilibrium between systemic venous hypertension and reduced cardiac output that predisposes patients to progressive morbidity [7,9]. With a prevalence of ~66 people per million (ppm) in developed countries and an estimated global population of just 50,000–70,000, Fontan circulation is a very rare condition [12,13]. Aside from heart transplantation, there are no evidence-based therapies currently available for treating patients with Fontan circulation.

Emerging data suggest that metabolic stress and mitochondrial dysfunction are integral to Fontan pathophysiology, yet the systemic lipidomic landscape remains undefined [14,15]. Given the metabolic demands of this unique physiology, plasma lipid profiles may offer insight into underlying bioenergetic status, end-organ stress, or adaptive changes. In this study, we integrated untargeted lipidomic profiling with detailed clinical, hemodynamic, and exercise performance data in adults with Fontan circulation and matched controls. We aimed to define the lipidomic signature of Fontan physiology, identify lipid species associated with cardiovascular performance, and evaluate their potential as a promising strategy to better understand Fontan pathophysiology.

## 2. Materials and Methods

### 2.1. Study Participants

Adult Fontan patients and age- and sex-matched healthy (no history of congenital heart disease or systemic disorders) controls (*n* = 20 per group) were prospectively recruited. Among the 20 Fontan patients, 14 had undergone extracardiac Fontan procedures, 4 had lateral tunnel repairs, 1 had a Bjork-type repair, and 1 had a classic atriopulmonary connection. The mean duration since Fontan completion in this cohort was 22 ± 6 years. Exclusion criteria for the Fontan group were New York Heart Association (NYHA) class IV symptoms, intellectual or physical disability limiting study participation, or being currently under work-up for heart transplantation. All participants underwent comprehensive assessments, including body composition, frailty evaluation, cardiopulmonary exercise testing (CPET), and non-invasive hemodynamic assessment, both at rest and post-exercise, using the Non-Invasive Cardiac System (NICaS) (NI medical, Israel) [16]. Detailed methodologies for these procedures have been described in our previously published metabolomics work using the same cohort [15].

Venous blood samples were collected from each participant in the fasting state in the morning. Samples were drawn into EDTA tubes and immediately centrifuged at 2500× *g* for 5 min at 4 °C. Plasma was separated, aliquoted into microcentrifuge tubes (MCT), and immediately stored at −80 °C until further analysis. The mean time from blood collection to storage was kept consistent and is usually less than 20 min. Informed consent was obtained from all subjects in this study before enrolment. The study was approved by the Research Ethics Boards of the University of Manitoba and St. Boniface Hospital.

### 2.2. Lipid Analysis

Before lipid extraction, the plasma samples were thawed for nearly 20 min. Lipids were extracted using a single-phase chloroform: methanol (2:1, *v*/*v*) protocol as described previously [17,18,19,20]. In brief, 10 µL of plasma was mixed with 200 µL of chloroform/methanol (2:1, *v*/*v*) and 30 μL of internal lipid standards (ISTD). This mixture was vortexed for 10 min and then subjected to 30 min of sonication in a water bath at room temperature (RT). The mixture was allowed to settle for 20 min at RT before being centrifuged for 20 min at 20,000× *g* at RT. The upper phase, which contained the lipids, was transferred into a clean 1.5 mL microcentrifuge tube and dried under a stream of nitrogen gas. The dried extracts were then redissolved in 50 μL of water-saturated 1-butanol, sonicated for 10 min, followed by the addition of 50 μL of 10 mM ammonium formate in methanol. The extract was vigorously shaken for 10 seconds and subsequently centrifuged at 10,000× *g* for 10 min at RT. Finally, 80 µL of the clean supernatant was collected for LC-MS analysis.

The samples were randomized prior to the mass spectrometry (MS) run. The separation and detection of the lipids were carried out on a Prominence chromatographic system (Shimadzu Corporation, Canby, OR, USA) paired with an AbSciex 4000 QTRAP triple quadrupole mass spectrometer (AB Sciex, Framingham, MA, USA). Detailed information on the analytical (targeted lipidomics) platform, including sample preparation, quality control measures, instrument parameters, and data processing, can be found in our previously published work [18].

### 2.3. Statistical Analysis

All statistical analyses and data visualization were performed using R language v4.4.2 [21]. Lipid abundance values were log_10_-transformed prior to statistical analysis to reduce skewness and improve comparability between lipid species. Group-wise comparisons of lipid abundances between Fontan and control subjects were performed using the Wilcoxon rank-sum test (Mann–Whitney U test). Significance was defined as *p* < 0.05. The lipid correlation network analysis was performed using the igraph R package(version 4.2.3) [22], and Lipid pathway enrichment analysis was conducted using LIPEA (Lipid Pathway Enrichment Analysis) [23], a web-based tool that maps lipid species to KEGG pathways and identifies statistically overrepresented lipid signatures. Pearson correlation coefficients and associated *p*-values were computed using the rcorr() function from the Hmisc R package [24]. Correlation matrices were visualized using the corrplot R package [25]. Discriminatory performance was evaluated using receiver operating characteristic (ROC) curve analysis. The area under the curve (AUC) was computed for each variable using the pROC package in R [26]. Confidence intervals (95%) for AUC were estimated using nonparametric bootstrap resampling (n = 5000 iterations). ROC curves were smoothed using binormal smoothing for selected lipid species.

## 3. Results

### 3.1. Clinical, Functional, and Hemodynamic Characteristics of Fontan Patients

Table 1 summarizes the clinical, cardiopulmonary, and hemodynamic profiles of adults with Fontan circulation compared to age- and sex-matched healthy controls. The two groups were similar in baseline characteristics, including age (25.5 vs. 30 years, *p* = 0.239), sex distribution (70% vs. 65% male), and BMI (24.1 vs. 26.1 kg/m^2^, *p* = 0.118). However, Fontan patients exhibited significant impairments in body composition parameters indicative of reduced metabolic reserve. Specifically, they had lower skeletal muscle mass (27.9 ± 6.3 vs. 34.3 ± 7.8 kg, *p* = 0.007), dry lean mass (13.5 ± 2.8 vs. 16.2 ± 3.5 kg, *p* = 0.009), lean body mass (50.8 ± 10.0 vs. 60.4 ± 12.8 kg, *p* = 0.012), and basal metabolic rate (1455.8 ± 228.8 vs. 1675.7 ± 277.4 kcal, *p* = 0.010). Although matched for chronological age, these findings indicate that Fontan patients have reduced metabolic reserve, which may influence systemic lipid metabolism independent of age.

Exercise capacity was markedly reduced in the Fontan patients, as demonstrated by significantly lower peak VO_2_ (23.7 ± 5.6 vs. 43.9 ± 8.6 mL/kg/min, *p* < 0.001), VO_2_max (1.7 ± 0.5 vs. 3.4 ± 0.9 L/min, *p* < 0.001), VO_2_max predicted (64.8 ± 14.0 vs. 124.3 ± 23.2%, *p* < 0.001), and VCO_2_ (1.8 ± 0.5 vs. 4.1 ± 1.1 L/min, *p* < 0.001), alongside an elevated VE/VCO_2_ ratio (39.3 ± 4.8 vs. 32.0 ± 3.6, *p* < 0.001), indicating ventilatory inefficiency.

At rest, Fontan patients demonstrated significantly lower stroke index (SI) (27.7 ± 7.9 vs. 45.6 ± 7.4 mL/m^2^, *p* < 0.001), cardiac index (CI) (2.1 ± 0.7 vs. 2.9 ± 0.5 L/min/m^2^, *p* < 0.001), cardiac output (CO) (3.9 ± 1.6 vs. 5.6 ± 1.1 L/min, *p* < 0.001), and cardiac power index (CPI) (0.4 ± 0.1 vs. 0.6 ± 0.1 W/m^2^, *p* < 0.001). Notably, the dynamic increases in CI, CPI, and CPO post-exercise (Δ hemodynamics) were also significantly blunted in Fontan subjects, reflecting impaired cardiovascular reserve.

### 3.2. Altered Plasma Lipid Signatures Associated with Fontan Physiology

**Figure 1:**
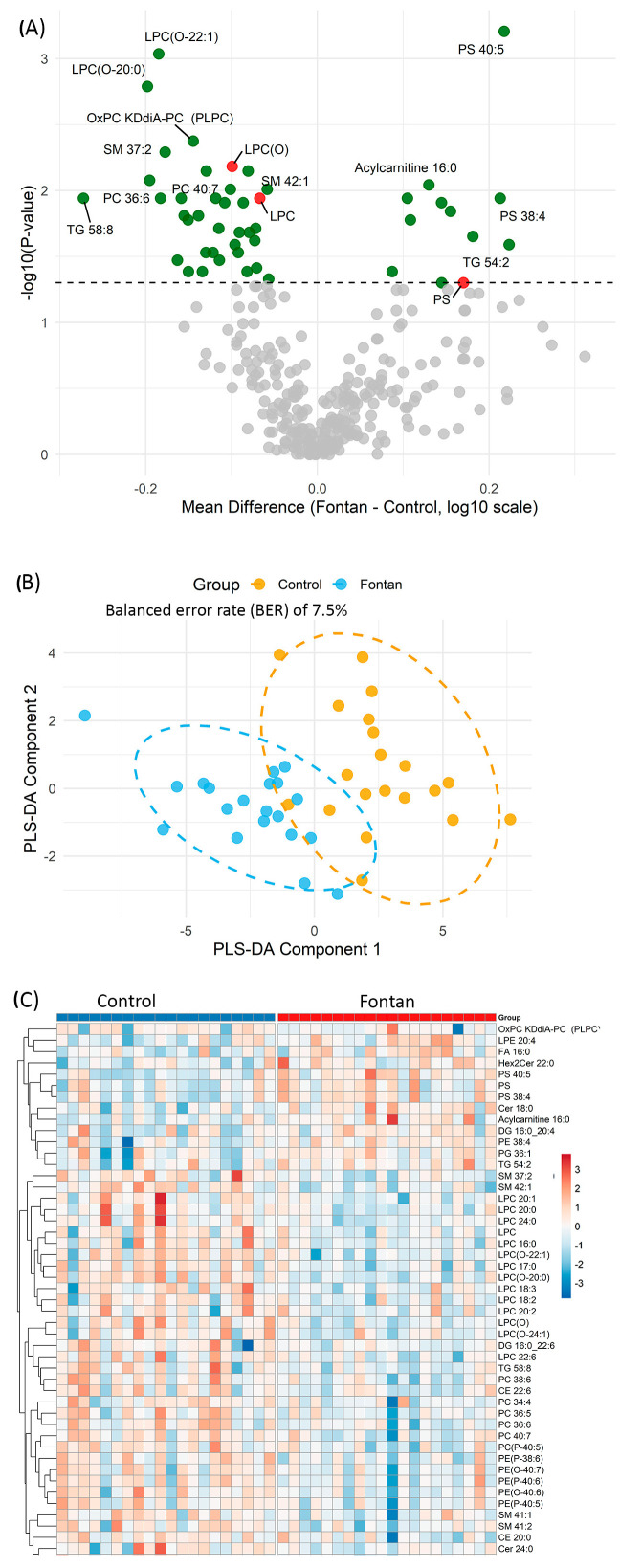
Lipidomic profiling distinguishes Fontan patients from controls. (**A**) Volcano plot showing differential plasma lipid expression. Each point represents a lipid species. The *x*-axis indicates the mean difference in log_10_-transformed abundance (Fontan—Control); the *y*-axis shows –log_10_ *p*-values from Wilcoxon rank-sum test. Lipids with *p* < 0.05 are highlighted in green; total lipid class amount with *p* < 0.05 are highlighted in red; selected significant species are annotated. (**B**) Partial least squares discriminant analysis (PLS-DA) plot based on lipids significantly altered between groups (*p* < 0.05). Each point is an individual sample, coloured by group. Ellipses denote 95% confidence regions. (**C**) Heatmap of hierarchically clustered lipid species differing between groups (*p* < 0.05). Columns represent individual samples; rows denote lipid species. Red indicates relative upregulation; blue indicates downregulation. **Abbreviations:** PLS-DA, partial least squares discriminant analysis; *p*, *p*-value.

Analysis of the plasma lipidome revealed several statistically significant differences between Fontan and control subjects (Figure 1A, Supplementary Table S1). Of the 291 lipid species quantified, 48 were significantly altered between groups (*p* < 0.05, Wilcoxon rank-sum test), including class-level differences in LPC, ether-linked LPC [LPC(O)], and PS, as well as individual ceramides (Cer) such as Cer 18:0 and Cer 24:0. Total LPC and LPC(O) levels were consistently decreased in the Fontan group, while total PS levels were significantly elevated relative to controls. At the species level, LPC(O-22:1) (*p* < 0.001, log_2_FC = −0.61) and LPC(O-20:0) (*p* = 0.002, log_2_FC = −0.66) were among the most significantly downregulated ether-linked LPCs. Conversely, PS 40:5 showed a marked increase (*p* < 0.001, log_2_FC = +0.72), reinforcing the overall class-level trends. Within the ceramide class, Cer 18:0 was significantly elevated (*p* = 0.014), whereas Cer 24:0 was reduced (*p* = 0.024), highlighting a complex reorganization of sphingolipid metabolism. These findings underscore coordinated alterations across both lipid classes and individual species that characterize the Fontan lipidomic phenotype.

To further assess the discriminatory capacity of the altered lipid profile, Partial Least Squares Discriminant Analysis (PLS-DA) was performed using the lipid species that differed significantly between groups. The resulting score plot (Figure 1B) displays the first two PLS components, with individual samples coloured by group (Fontan or Control) and ellipses representing the 95% confidence intervals. The PLS-DA model achieved a classification error rate of 7.5% and a balanced error rate (BER) of 7.5% on the first component based on 10-fold cross-validation, indicating strong separation between groups based on plasma lipidomic signatures.

Unsupervised hierarchical clustering of the same 48 significantly altered lipid species further demonstrated clear group-level separation between Fontan and control subjects (Figure 1C). Each row in the heatmap represents a lipid species and each column an individual subject, with lipid abundance values log_10_-transformed and standardized (Z-score). Subjects clustered predominantly according to clinical group, indicating coordinated lipidomic changes associated with Fontan physiology. A distinct cluster of LPC and LPC(O) species—including LPC 16:0, LPC 18:2, LPC 18:3, LPC 20:0, LPC(O-20:0), and LPC(O-22:1)—exhibited consistently lower abundance in the Fontan group, suggesting widespread suppression of these membrane-derived lipids. In contrast, elevated levels of specific PS and ceramide species—including PS 40:5, PS 38:4, and Cer 18:0—were observed in the Fontan group, with these lipids clustering predominantly among Fontan subjects in the heatmap. Together, these findings from univariate, multivariate, and clustering analyses consistently demonstrate that the Fontan population exhibits a distinctive plasma lipidomic signature, marked by suppression of LPC-related species and upregulation of PS and ceramides.

### 3.3. Lipid Correlation Network Reveals Class-Specific Clustering

**Figure 2:**
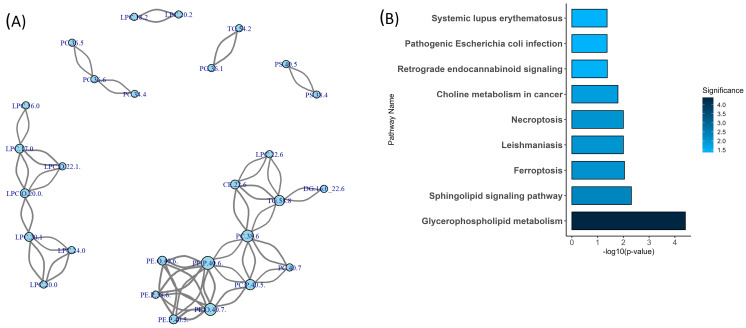
Network connectivity and pathway enrichment of differentially expressed lipid species in Fontan circulation. (**A**) Correlation network of significantly altered lipids. Nodes represent individual lipid species; edges denote strong positive or negative Pearson correlations (|*r*| > 0.7). Node size reflects connectivity (degree), and edge width corresponds to correlation strength. Distinct lipid clusters suggest coordinated metabolic regulation. (**B**) Lipid pathway enrichment analysis. Bar plot shows significantly enriched lipid metabolic pathways (*p* < 0.05) identified using the LIPEA platform. Bar colour intensity corresponds to pathway significance (darker = more significant). **Abbreviations:** LIPEA, Lipid Pathway Enrichment Analysis; *p*, *p*-value; *r*, Pearson correlation coefficient.

Lipid correlation network analysis was employed to explore co-regulatory patterns among the altered lipid species, using a threshold of |r| > 0.7 to define strong correlations. This network-based approach enabled the identification of lipid modules that co-varied across samples, reflecting distinct patterns of association. The resulting network (Figure 2A) revealed three prominent and discrete clusters: (1) an LPC/LPC(O) cluster, comprising species such as LPC(16:0), LPC(17:0), LPC(18:2), LPC(20:0), LPC(O-20:0), and LPC(O-22:1), which exhibited moderate to strong intercorrelations; (2) a PE cluster, dominated by ether-linked species including PE(P-40:6) and PE(O-40:7), characterized by high internal connectivity; and (3) a PS cluster (e.g., PS 38:4, PS 40:5), which appeared sparsely connected and distinct from other lipid groups.

To complement the network analysis, Lipid Pathway Enrichment Analysis [23] (LIPEA) was performed (Figure 2B) using annotations from the Kyoto Encyclopedia of Genes and Genomes (KEGG). This analysis identified two significantly enriched pathways among the altered lipid species: glycerophospholipid metabolism (*p* < 0.001) and the sphingolipid signalling pathway (*p* = 0.005). The glycerophospholipid metabolism pathway included several of the most connected lipid species, such as LPC(16:0), LPC(17:0), and PE(P-40:6). The sphingolipid signalling pathway involved lipids such as SM(42:1) and Cer(24:0). Together, these results highlight class-specific clustering patterns and pathway-level associations among the altered lipid species.

### 3.4. Relationship Between Exercise Capacity and Hemodynamic Performance

In a pooled cohort of both Fontan and healthy subjects, Pearson correlation revealed strong association between exercise and hemodynamic parameters as expected (Supplementary Figure S1). For example, VO_2_max was strongly associated with CPO post-exercise (r = 0.77, *p* < 0.001) and SI at rest (r = 0.76, *p* < 0.001). These relationships reflect a physiologically coherent link between aerobic capacity and cardiac performance, both at baseline and following exertion. While post-exercise indices showed the highest correlations, delta measures such as CI change and CPI change also trended positively with VO_2_max and VCO_2_, though at a more modest magnitude. This may reflect the variability in cardiovascular reserve. Together, these findings suggest that resting and post-exercise hemodynamic markers are strongly aligned with cardiorespiratory performance and may serve as useful surrogates of functional capacity in this population.

### 3.5. Lipid Species Associated with Exercise Capacity in Fontan Patients

**Figure 3:**
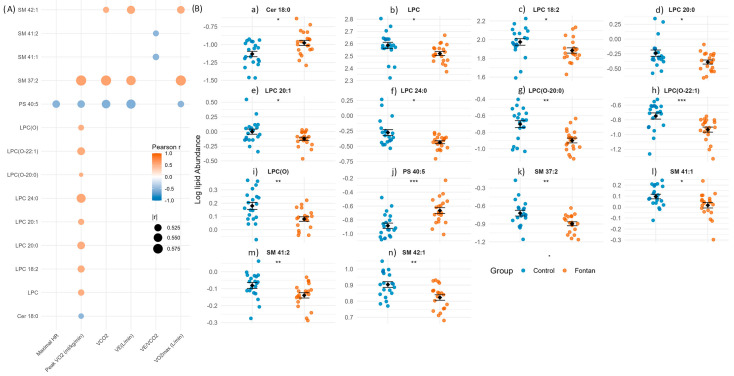
Lipid species associated with exercise capacity in Fontan patients. (**A**) Correlation map of lipid species significantly altered between Fontan and control groups (*p* < 0.05, Wilcoxon rank-sum test) and strongly correlated with at least one exercise parameter (|*r*| > 0.5, *p* < 0.05, Pearson). Lipids are plotted on the *y*-axis and exercise variables on the *x*-axis. Dot colour reflects correlation direction and strength; circle size indicates magnitude (|*r*|). (**B**) Group-wise comparison of correlated lipid species. Dot plots show individual lipid abundances with mean ± SEM overlaid. Statistical comparisons were performed using the Wilcoxon rank-sum test; significance is denoted as: *p* < 0.05 (*), *p* < 0.01 (**), *p* < 0.001 (***). **Abbreviations:** *p*, *p*-value; *r*, Pearson correlation coefficient; SEM, standard error of the mean; Maximal HR, maximal heart rate; Peak VO_2_, peak oxygen uptake normalized to body weight; VCO_2_, carbon dioxide production; VE, minute ventilation; VE/VCO_2_, ventilatory equivalent for carbon dioxide; VO_2_max, maximal oxygen uptake.

Among the lipid species significantly altered between Fontan patients and controls (Wilcoxon rank-sum test, *p* < 0.05), LPC, LPC(O), SM, PS, and Cer showed strong and directionally distinct associations with exercise performance (Figure 3A). Correlation analysis revealed that LPC, LPC(O), and SM species were positively associated with Peak VO_2_ and VO_2_max (Pearson r > 0.50, *p* < 0.001). In contrast, PS 40:5 and Cer 18:0 exhibited strong negative correlations with the same exercise parameters (r < –0.50, *p* < 0.001). In addition, linear regression analysis assessing the association between significantly correlated plasma lipid species and exercise physiology parameters, adjusted for Skeletal Muscle Mass (Supplementary Table S2), showed that all these associations remained statistically significant after adjusting for skeletal muscle mass (SMM), suggesting that these lipid–exercise relationships are independent of body composition.

To further explore these relationships, we generated dot plots (Figure 3B) comparing the abundance of strongly correlated lipid species between Fontan and control groups. Fontan patients exhibited significantly lower levels of LPC, LPC(O), and SM species, and higher levels of PS and Cer species, consistent with the observed correlations. These findings underscore the relevance of specific lipid alterations not only as markers of disease phenotype but also as potential indicators of exercise capacity and cardiometabolic adaptation in the Fontan circulation.

### 3.6. Lipid Profiles Reflect Hemodynamic Function and Adaptability

**Figure 4:**
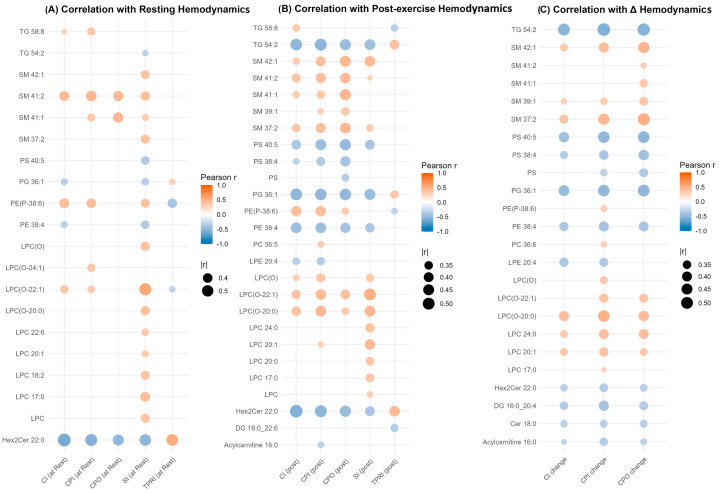
Correlation of significantly altered lipid species with hemodynamic parameters in Fontan physiology. Dot plots show Pearson correlation coefficients (colour-coded) between lipid species significantly altered between Fontan patients and controls (*p* < 0.05, Wilcoxon rank-sum test) and key hemodynamic parameters measured at rest (Panel (**A**)), post-exercise (Panel (**B**)), and as delta values (Panel (**C**)). Lipids are displayed on the *y*-axis, and hemodynamic variables on the *x*-axis. Dot size reflects the strength of correlation (|r| > 0.3), and colour represents correlation direction and magnitude (blue = negative, red = positive). Only statistically significant correlations (*p* < 0.05) are shown. **Abbreviations**: CI, cardiac index; CPI, cardiac power index; CPO, cardiac power output; SI, stroke index; and TPRI, total peripheral resistance index.

To explore the relationship between lipid metabolism and cardiovascular performance in Fontan physiology, we examined correlations between significantly altered lipid species (Wilcoxon *p* < 0.05) and hemodynamic indices measured at rest, post-exercise, and the exercise-augmented changes (Δ). Although lipidomic measurements were obtained from resting plasma, hemodynamic data were available for all three states (Figure 4A–C). Several phospholipids and sphingolipids demonstrated moderate to strong correlations (|r| > 0.3) with key parameters, including SI, CI, and CPI.

Among the most consistently associated species were LPC 20:1, ether-linked LPC(O) lipids such as LPC(O-20:0) and LPC(O-22:1), and Hex2Cer 22:0. LPC and LPC(O) species showed positive correlations with CI and SI at rest, many of which persisted post-exercise and with Δ values. For instance, LPC(O-20:0) and LPC(O-22:1) were positively correlated with CI and SI across all physiological states, including ΔCI and ΔCPI. In contrast, Hex2Cer 22:0 exhibited strong negative correlations with CI and CPI at rest (r < –0.5, *p* < 0.001), with similar trends post-exercise and in Δ values, indicating a reproducible inverse association with cardiac output and performance. Notably, the directionality of associations remained consistent within lipid classes: LPC and LPC(O) species correlated positively with indices of cardiac function, while Hex2Cer species exhibited inverse relationships. Taken together, these results identify a small subset of lipid species, particularly LPC, LPC(O), and Hex2Cer-based molecules, that demonstrate reproducible associations with multiple measures of cardiovascular function across rest, exercise, and adaptive change.

### 3.7. Discriminatory Performance of Clinical and Lipidomic Variables

**Figure 5:**
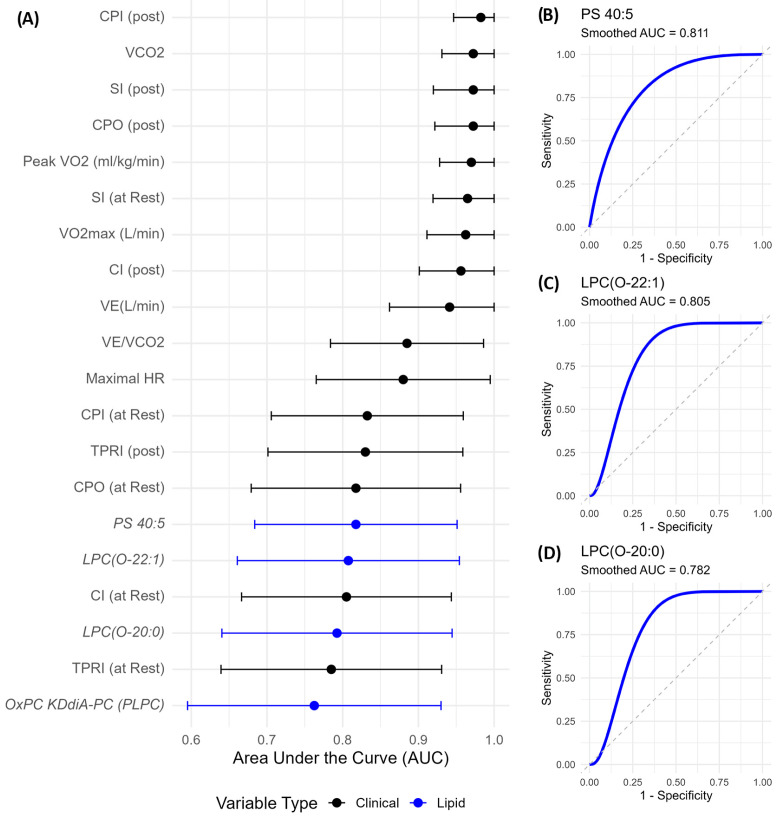
Discriminatory performance of clinical and lipidomic variables distinguishing Fontan from control subjects. (**A**) Top 20 variables ranked by AUC with 95% bootstrap confidence intervals. Lipid species (blue) include PS 40:5, LPC(O-22:1), and LPC(O-20:0), which showed strong discriminatory performance. (**B**–**D**) Smoothed ROC curves for the top lipid markers. AUC values are annotated. Ether-linked LPC and PS species reflect biologically relevant lipid remodelling in Fontan circulation. **Abbreviations**: AUC, area under the curve; ROC, receiver operating characteristic; PS, phosphatidylserine; LPC(O), ether-linked lysophosphatidylcholine.

To evaluate the discriminatory capacity of clinical, exercise, hemodynamic, and lipidomic variables between Fontan patients and controls, we performed receiver operating characteristic (ROC) curve analysis and computed area under the curve (AUC) values with 95% confidence intervals using bootstrap resampling (n = 5000). Post-exercise hemodynamic parameters emerged as the strongest classifiers (Figure 5A). Post-exercise CPI (CPI post) showed the highest discriminative power (AUC = 0.983, 95% CI: 0.947–1.000), followed closely by VCO_2_, post-exercise SI, and CPO (all AUCs ≥ 0.972).

Among lipid species, PS 40:5 showed the highest AUC (0.818, Figure 5B), followed by LPC(O-22:1) (AUC = 0.805, Figure 5C) and LPC(O-20:0) (AUC = 0.782, Figure 5D). Notably, these lipid species performed comparably to or better than resting hemodynamic parameters, underscoring their potential as non-invasive, biologically meaningful biomarkers. While their confidence intervals were wider than those of clinical indices, reflecting biological heterogeneity, their lower bounds remained well above 0.5, indicating consistent discriminatory value.

## 4. Discussion

In this study, we present the first comprehensive characterization of the plasma lipidomic landscape in adults with Fontan circulation, integrating untargeted lipid profiling with clinical, hemodynamic, and exercise performance data. Our findings reveal a distinct lipidomic signature in Fontan patients compared to age- and sex-matched clinically healthy controls, characterized by a consistent reduction in total LPC and LPC(O), and a concomitant increase in total PS levels. At the species level, several LPC species including LPC 16:0, LPC 18:2, and LPC 20:0, as well as LPC(O-20:0) and LPC(O-22:1), were significantly downregulated in Fontan plasma. In contrast, PS species such as PS 40:5 and PS 38:4 were markedly elevated. These lipid alterations demonstrated strong associations with key physiological measures, including VO_2_max and SI. Select species from these two classes, particularly LPC(O-20:0), LPC(O-22:1) and PS 40:5, also showed discriminatory performance comparable to established clinical indices. Collectively, these findings highlight coordinated lipid remodelling in Fontan circulation, characterized by suppression of LPC-related species and upregulation of PS, which may reflect or contribute to the hemodynamic and functional limitations observed in this physiologically complex population.

The reduction in LPC is consistent with prior observations in cardiovascular diseases [27,28,29], rheumatoid arthritis [30], pulmonary arterial hypertension [31], and liver cirrhosis [32] where reduced LPC levels have been linked to increased mortality risk [33]. It has been reported that, in cancer patients, decreased LPC levels were associated with weight loss and increased inflammation, and is an indicator of disease severity [34]. LPCs are predominantly produced through hepatic phospholipase A2 activity on PC [35,36] or via lecithin-cholesterol acyltransferase (LCAT)-mediated remodelling in plasma [37]. In the Fontan circulation, hepatic congestion and dysfunction may impair LPC synthesis and release. Additionally, chronic inflammation and oxidative stress commonly observed in this population [38] may accelerate the metabolic turnover or degradation of circulating LPCs.

Prior lipidomic studies have reported associations between reduced ether lipid levels with hypertension [39], and age [40]. Ether-linked LPC(O) species are synthesized via the peroxisomal ether lipid biosynthetic pathway [41], and their depletion may reflect impaired peroxisomal function or mitochondrial–peroxisomal dysregulation. Supporting this notion, we also observed elevated levels of acylcarnitine 16:0 (palmitoylcarnitine), a biomarker of incomplete mitochondrial β-oxidation and impaired fatty acid catabolism [42]. The concurrent suppression of LPC(O) and accumulation of long-chain acylcarnitines suggests coordinated mitochondrial–peroxisomal dysfunction in Fontan physiology. LPCs are known to act as homeostatic mediators in vascular inflammation through modulation of endothelial activation and leukocyte infiltration [33]. Their suppression, alongside markers of impaired energy metabolism, may contribute to the pro-inflammatory milieu, endothelial dysfunction, and reduced aerobic efficiency, which are hallmarks of Fontan circulation.

Lipid correlation network analysis revealed distinct modular clustering among LPC/LPC(O), and PS species. This class-specific organization suggests coordinated regulation of lipid metabolism under Fontan physiology and may reflect shared enzymatic or signalling pathways within each lipid module. Within the LPC/LPC(O) cluster, species including LPC 16:0, LPC 17:0, LPC 20:0, LPC 20:1, LPC 24:0, LPC(O-20:0), and LPC(O-22:1), formed a tightly connected correlation cluster, suggesting coordinated regulation. Pathway enrichment identified these species as part of the glycerophospholipid metabolism pathway, highlighting a shared metabolic origin and reinforcing the biological coherence of these findings.

Interestingly, even though LPC and LPC(O) levels were suppressed in Fontan plasma, these species demonstrated consistent positive associations with cardiovascular function across physiological states. In particular, LPC 20:1, LPC(O-20:0), and LPC(O-22:1) were positively correlated with peak VO_2_, and SI, at rest, and post-exercise. These relationships suggest that higher circulating levels of these ether-linked lipids are associated with preserved myocardial function and improved circulatory reserve. Moreover, LPC(O-20:0) and LPC(O-22:1) demonstrated strong discriminatory performance (AUC = 0.782 and 0.805, respectively), performing comparably or better than resting hemodynamic indices in distinguishing Fontan patients from controls. Collectively, these results support the utility of LPC and LPC(O) species as non-invasive indicators of circulatory health and functional capacity in Fontan physiology. Although our cohort size (*n* = 20 per group) is reasonable given the rarity of Fontan circulation, statistical power remains limited. Larger multicentre collaborations or pooled analyses across international cohorts will be essential to confirm these findings and evaluate their prognostic utility.

In contrast, PS levels were significantly elevated in Fontan patients, both at the class and species level. PS 40:5 and PS 38:4 showed the most marked increases and were inversely correlated with VO_2_max, indicating a link between PS enrichment and reduced aerobic capacity. PS is a negatively charged phospholipid normally localized to the inner leaflet of the cell membrane but becomes externalized during apoptosis, oxidative stress, and inflammatory activation [43]. Elevated circulating PS may therefore reflect increased apoptosis [14], or chronic inflammation [38], each of which is a recognized feature of Fontan physiology. Similar PS enrichment has been documented in chronic heart failure (HF), where PS enriched microparticles contributes to the procoagulant state in HF patients [44]. In our study, PS 40:5 was inversely related to VO_2_max, suggesting that PS dysregulation may be linked to impaired ventilatory efficiency and reduced aerobic capacity. These findings raise the possibility that elevated PS levels may not only reflect underlying circulatory stress but also actively contribute to adverse vascular tone and reduced aerobic capacity in Fontan physiology.

From a translational perspective, while LPC(O-22:1), LPC(O-20:0), and PS 40:5 emerged as promising candidate biomarkers, several feasibility considerations warrant attention. Standardized assays for these ether-linked phospholipids and PS species are not yet widely available in clinical laboratories, and specialized mass spectrometry platforms remain costly and require technical expertise. Reproducibility across laboratories and platforms, as well as pre-analytical factors such as sample collection, processing, and storage, can also affect lipid measurements and must be rigorously standardized before clinical application. Future work should focus on validating these markers across independent cohorts, developing streamlined targeted assays, and evaluating their incremental value relative to conventional clinical and biochemical parameters.

Our current lipidomic findings align with and expand upon our previous investigation of bile acid (BA) dysregulation in the same Fontan cohort, where we observed elevated circulating BA levels in Fontan patients [15]. In animal models, BAs have been shown to induce oxidative stress and disrupt mitochondrial integrity in hepatocytes [45]. Their elevated levels can also impair cardiac mitochondrial function by promoting mitochondrial permeability transition [46]. These effects are particularly relevant in Fontan physiology, which is marked by hepatic congestion, chronic low cardiac output, and systemic inflammation [4]. In this study, we additionally observed suppression of LPC and LPC(O) species, and elevation of acylcarnitine 16:0, a biomarker of incomplete mitochondrial fatty acid oxidation. Together, these findings point to a common pathway of mitochondrial metabolic stress, whereby mitochondrial dysfunction and systemic lipid remodelling converge to impair energy production and cardiovascular reserve in Fontan circulation. Moreover, the upregulation of phosphatidylserines (PS), often associated with apoptosis and inflammatory signalling, reinforces the presence of a pro-inflammatory and energetically compromised systemic state. Together, elevated BAs, reduced LPCs, increased PS, and disrupted mitochondrial lipid homeostasis characterize an integrated metabolic profile. This reflects the multisystem metabolic burden of Fontan circulation and underscores the potential for targeting mitochondrial and lipid signalling pathways in future therapeutic strategies.

Our study was cross-sectional in design, which precludes causal inference between lipidomic alterations and functional outcomes such as VO_2_max or cardiac index. Moreover, because lipidomics was performed on resting plasma samples only, the analysis does not capture dynamic changes in lipid metabolism during exercise or longitudinal disease progression. Future studies incorporating serial sampling, exercise-challenge protocols, and longitudinal follow-up will be necessary to determine whether these lipid signatures are stable markers of disease state, responsive to physiological stress, or predictive of adverse outcomes. Another limitation is that potential confounding factors such as dietary intake, medication use (e.g., statins, ACE inhibitors), and systemic inflammatory status were not systematically captured in this study. Each of these factors can influence circulating lipid levels and may contribute to the variability observed in our dataset. While the matched control design mitigates some of this concern, future studies incorporating detailed nutritional, pharmacological, and biochemical profiling will be important to disentangle the effects of underlying physiology from modifiable external influences. Because none of the lipid species remained statistically significant after correction for multiple comparisons, our conclusions are based on unadjusted *p*-values and should be considered exploratory.

## 5. Conclusions and Perspectives

This study provides the first comprehensive lipidomic characterization of adults with Fontan circulation, revealing a distinct metabolic signature marked by coordinated reductions in LPC and LPC(O), and elevations in PS and selected ceramide species. These lipid alterations were not only statistically robust but also biologically meaningful, correlating strongly with key clinical and hemodynamic metrics. The integration of lipidomic profiling with cardiovascular performance highlights the potential of specific lipid species, particularly LPC(O-22:1), LPC(O-20:0), and PS 40:5, as non-invasive biomarkers reflective of functional reserve in this physiologically complex population. The observed lipid changes together with prior evidence of bile acid dysregulation and mitochondrial dysfunction in the same cohort, underscore a broader theme of metabolic stress and remodelling in Fontan physiology. While exploratory, these results provide a foundation for future studies aimed at validating lipid-based biomarkers and investigating targeted metabolic interventions to improve long-term cardiovascular outcomes in Fontan patients.

## 6. Study Limitations

This study has several limitations that warrant consideration. First, the sample size was modest (*n* = 20 per group), which, while sufficient to detect class-level and species-level lipid alterations, may limit the statistical power to perform robust subgroup analyses. However, this reflects the rarity of Fontan physiology, which is estimated to occur in only 0.08 to 0.4 per 1000 live births [47], making the recruitment of well-characterized and age- and sex-matched cohorts inherently challenging. Due to the small sample size and the large number of comparisons, none of the lipid species remained statistically significant after correction for multiple testing; therefore, results are presented based on raw *p*-values and should be interpreted as exploratory and hypothesis-generating. Second, the cross-sectional design prevents causal inference and does not address temporal or exercise-induced changes in lipidomic profiles. Longitudinal and interventional studies will be required to clarify the trajectory and clinical implications of these lipid alterations. Lastly, potential confounders including diet, medications, and gut microbiota were not systematically controlled, and these may have influenced lipidomic signatures.

## Figures and Tables

**Table 1:** Clinical characteristics of the study population.

Clinical Variable	Fontan (n = 20)	Control (n = 20)	*p* Value
Age (years)	25.50 [22.75–30.25]	30.00 [25.75–34.25]	0.239
Sex (male)	14 (70.0%)	13 (65.0%)	1.000
Body mass index (kg/m^2^)	24.14 ± 4.47	26.12 ± 3.22	0.118
Body Surface Area (BSA)	1.80 ± 0.22	1.92 ± 0.21	0.099
Total body water (L)	36.80 ± 7.77	44.23 ± 9.37	0.010
Dry Lean Mass (kg)	13.46 ± 2.82	16.21 ± 3.47	0.009
Body fat (%)	27.49 ± 9.75	22.67 ± 8.34	0.101
Skeletal Muscle Mass (kg)	27.88 ± 6.34	34.30 ± 7.81	0.007
Lean Body Mass(kg)	50.77 ± 10.02	60.44 ± 12.84	0.012
Basal metabolic rate (kcal)	1455.80 ± 228.77	1675.70 ± 277.38	0.010
5 m walk (seconds)	3.87 ± 0.54	4.14 ± 0.65	0.158
**Cardiopulmonary exercise test**			
Maximum heart rate	157.50 [138.00–171.25]	185.00 [182.25–194.00]	<0.001
VO_2_max (L/min)	1.67 ± 0.46	3.43 ± 0.93	<0.001
Peak VO_2_ (ml/kg/min)	23.73 ± 5.64	43.88 ± 8.63	<0.001
VE(L/min)	68.25 ± 17.76	129.15 ± 35.40	<0.001
VCO_2_	1.76 ± 0.51	4.07 ± 1.14	<0.001
VE/VCO_2_	39.34 ± 4.75	31.99 ± 3.55	<0.001
**Hemodynamics—rest**			
SI	27.65 ± 7.95	45.55 ± 7.40	<0.001
CI	2.13 ± 0.74	2.96 ± 0.50	<0.001
CO	3.90 ± 1.59	5.65 ± 1.14	<0.001
CPI	0.40 ± 0.16	0.60 ± 0.12	<0.001
CPO	0.74 ± 0.34	1.15 ± 0.27	<0.001
TPRI	3162.00 [2682.75–3875.00]	2503.50 [2148.75–2781.75]	<0.001
**Hemodynamics—post-exercise**			
SI	27.90 ± 7.66	46.95 ± 8.02	<0.001
CI	2.79 ± 0.81	4.92 ± 1.05	<0.001
CO	4.95 [3.58–5.55]	9.05 [7.75–10.67]	<0.001
CPI	0.57 ± 0.18	1.21 ± 0.28	<0.001
CPO	1.04 [0.77–1.17]	2.22 [1.82–2.79]	<0.001
TPRI	2863.20 ± 978.18	1894.45 ± 475.29	<0.001
**Δ Hemodynamics**			
CI change	0.66 ± 0.69	1.96 ± 0.86	<0.001
CPI change	0.17 ± 0.14	0.61 ± 0.26	<0.001
CPO change	0.30 ± 0.24	1.16 ± 0.52	<0.001

Continuous variables are presented as mean ± standard deviation if normally distributed or as median [interquartile range] if non-normally distributed (based on Shapiro–Wilk test), while categorical variables are shown as counts with percentages. Statistical comparisons were performed using independent t-tests for normally distributed variables, Mann–Whitney U tests for non-normal variables, and Fisher’s Exact tests for categorical variables. Abbreviations: VO_2_max (Maximal Oxygen Consumption), VE (Minute Ventilation), VCO_2_ (Carbon Dioxide Production), VE/VCO_2_ (Ventilatory Equivalent for Carbon Dioxide), SI (Stroke Index), CI (Cardiac Index), CO (Cardiac Output), CPI (Cardiac Power Index), CPO (Cardiac Power Output), TPRI (Total Peripheral Resistance Index), and Δ CI/CPI/CPO (Change in respective hemodynamic parameters from rest to post-exercise). *p*-values < 0.05 were considered statistically significant. *p*-values are unadjusted and provided for descriptive purposes only.

## Data Availability

The raw LC–MS data files are publicly available in the MetaboLights repository under accession number MTBLS3839 (https://www.ebi.ac.uk/metabolights/MTBLS3839, accessed on 1 August 2025). Within the dataset, control samples are labelled with the prefix “Fon-Cnt” (metadata column Treatment = “control”), and Fontan samples are labelled with the prefix “Fontan” (metadata column Treatment = “Fontan procedure”).

## Supplementary Materials

The following supporting information can be downloaded at: https://www.mdpi.com/article/doi/s1, Figure S1: Correlation heatmap of clinical and functional parameters; Table S1: Differentially Abundant Lipid Species Between Fontan Patients and Controls Based on Wilcoxon Rank-Sum Test; Table S2: Lipid–Exercise Associations Adjusted for Skeletal Muscle Mass
