# Supplementary material for: Plasma Lipidomic Alterations in Fontan Circulation Reflect Cardiovascular Functional Reserve
Source: Metabolites. 2025 Sep 7;15(9):592. doi: 10.3390/metabo15090592 (PMC12471559; doi:10.3390/metabo15090592)
Supplement: Supplementary file 1 [file metabolites-15-00592-s001.zip › metabolites-3813262-Supplementary.pdf]

Plasma Lipidomic Alterations in Fontan Circulation Reflect Cardiovascular Functional Reserve.

Arun Surendran PhD[1], and Amir Ravandi MD PhD[2,3,4,5], Ashish H. Shah MD[3,4,5*]

[1]Mass Spectrometry Core Facility, BRIC-Rajiv Gandhi Centre for Biotechnology (RGCB), Thiruvananthapuram, Kerala, India; [2]Cardiovascular Lipidomics Laboratory, St. Boniface Hospital, Albrechtsen Research Centre, Manitoba, Canada; [3]Department of Physiology and Pathophysiology, Rady Faculty of Health Sciences, University of Manitoba, Manitoba, Canada; [4]Section of Cardiology, Department of Internal Medicine, Rady Faculty of Health Sciences, University of Manitoba, Manitoba, Canada; [5]Precision Cardiovascular Medicine Group, St. Boniface Hospital Research, Manitoba, Canada

***Corresponding author:** Dr. Ashish H. Shah
St Boniface Hospital
Winnipeg, MB, R2H 2A6
Canada
E-mail: Ashish.Shah@umanitoba.ca
Phone: +1 204 237 2023
Fax: +1 204 233 2157

Keywords: Fontan circulation, Lipidomics, LPC (Lysophosphatidylcholine), LPC(O) [ether-linked LPC], PS (phosphatidylserines), Cardiovascular physiology

Running Title: Fontan Circulation and lipidomics

Funding support: Establishment Grants.

Supplementary Figure S1

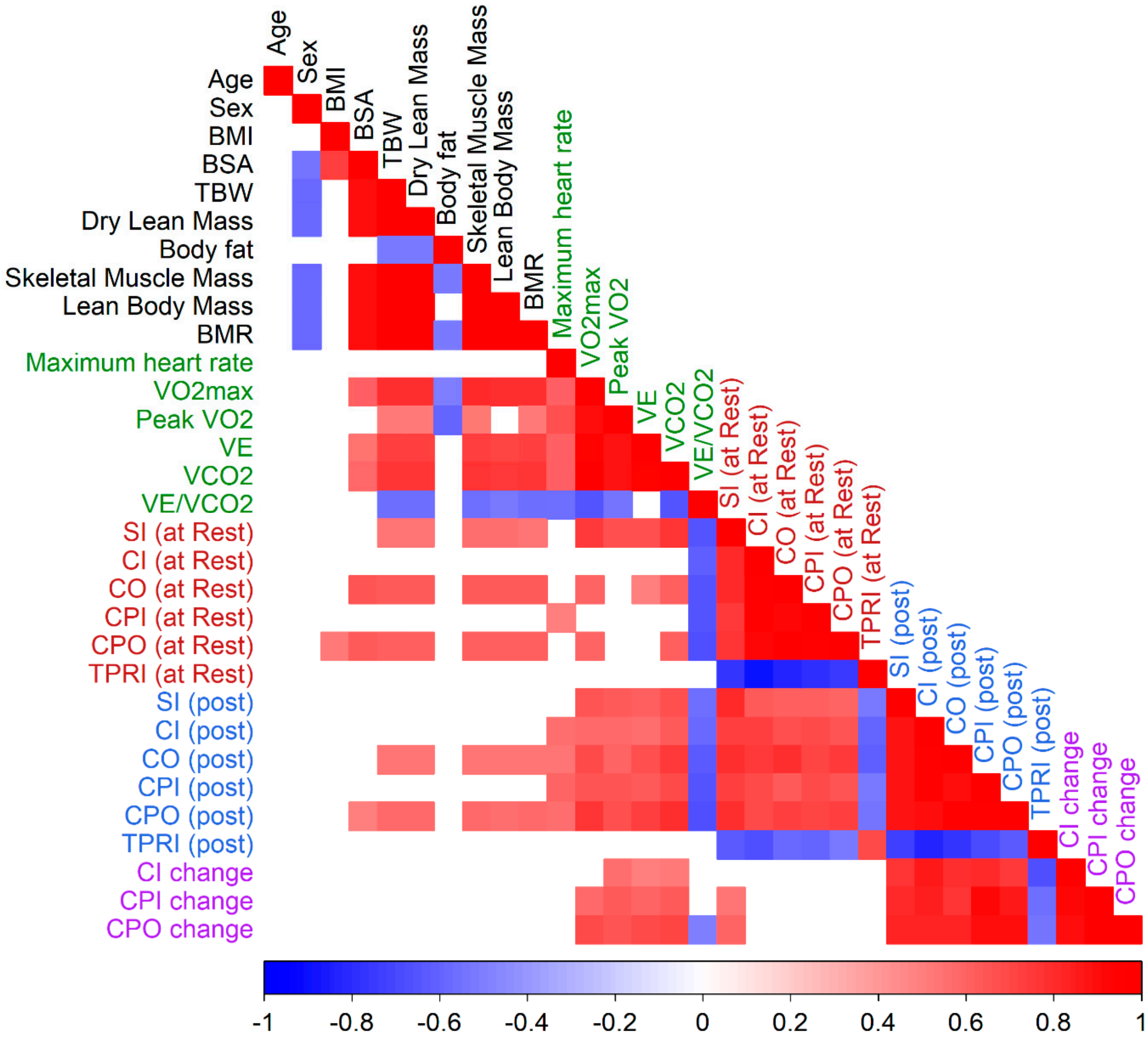

Supplementary Figure S1. Correlation heatmap of clinical and functional parameters. Heatmap showing significant and strong Pearson correlations ($|r| > 0.5$, $p < 0.05$) between exercise capacity measures and hemodynamic indices at rest, post-exercise, and as Δ (delta) changes in Fontan and control subjects ($n = 40$). Weak and non-significant associations are masked. Axis label colors indicate variable categories (e.g., exercise, rest, post-exercise, Δ change). **Abbreviations:** r, Pearson correlation coefficient; p, p-value; change, post–rest change; BMI, body mass index; BSA, body surface area; TBW, total body water; BMR, basal metabolic rate; VO_2max, maximal oxygen uptake; VE, minute ventilation; VCO_2, carbon dioxide production; VE/VCO_2, ventilatory equivalent for carbon dioxide; SI, stroke index; CI, cardiac index; CO, cardiac output; CPI, cardiac power index; CPO, cardiac power output; TPRI, total peripheral resistance index.

Supplementary Table S1: Differentially Abundant Lipid Species Between Fontan Patients and Controls Based on Wilcoxon Rank-Sum Test				
Sl. No	Lipid	p-value	B-H p-value	Log2_FC
1	LPC	0.011	0.177	-0.2233
2	LPC(O)	0.007	0.177	-0.3292
3	PS	0.05	0.325	0.5656
4	Cer 18:0	0.014	0.195	0.5147
5	Cer 24:0	0.024	0.229	-0.242
6	Hex2Cer 22:0	0.012	0.177	0.4804
7	SM 37:2	0.005	0.177	-0.5893
8	SM 39:1	0.019	0.21	-0.2388
9	SM 41:1	0.021	0.211	-0.2637
10	SM 41:2	0.01	0.177	-0.1937
11	SM 42:1	0.007	0.177	-0.2674
12	PC 34:4	0.017	0.195	-0.5
13	PC 36:5	0.034	0.266	-0.5414
14	PC 36:6	0.008	0.177	-0.6492
15	PC 38:6	0.016	0.195	-0.4596
16	PC 40:7	0.007	0.177	-0.4292
17	PC(P-40:5)	0.029	0.245	-0.3057
18	LPC 16:0	0.047	0.321	-0.1881
19	LPC 17:0	0.012	0.177	-0.3586
20	LPC 18:2	0.021	0.211	-0.3021
21	LPC 18:3	0.041	0.289	-0.2721
22	LPC 20:0	0.041	0.289	-0.4987
23	LPC 20:1	0.029	0.245	-0.4037
24	LPC 20:2	0.038	0.289	-0.2351
25	LPC 22:6	0.019	0.21	-0.3812
26	LPC 24:0	0.011	0.177	-0.5266
27	LPC(O-20:0)	0.002	0.171	-0.6581
28	LPC(O-22:1)	0.001	0.146	-0.6138
29	LPC(O-24:1)	0.012	0.177	-0.2872
30	PE 38:4	0.017	0.195	0.3599
31	PE(O-40:6)	0.01	0.177	-0.3367
32	PE(O-40:7)	0.034	0.266	-0.3791
33	PE(P-38:6)	0.011	0.177	-0.6068
34	PE(P-40:5)	0.026	0.232	-0.3188
35	PE(P-40:6)	0.011	0.177	-0.3934
36	LPE 20:4	0.011	0.177	0.3484
37	PS 38:4	0.011	0.177	0.7075

38	PS 40:5	0.001	0.146	0.7226
39	PG 36:1	0.022	0.22	0.6014
40	CE 20:0	0.029	0.245	-0.4312
41	CE 22:6	0.041	0.289	-0.4449
42	Acylcarnitine 16:0	0.009	0.177	0.4317
43	OxPC KDdiA-PC (PLPC)	0.004	0.177	-0.4807
44	DG 16:0_20:4	0.05	0.325	0.4808
45	DG 16:0_22:6	0.016	0.195	-0.515
46	TG 54:2	0.026	0.232	0.7417
47	TG 58:8	0.011	0.177	-0.9052
48	FA 16:0	0.041	0.289	0.2895

This table presents the results of the Wilcoxon rank-sum test comparing plasma lipid levels between Fontan patients and age- and sex-matched controls. For each lipid species, the raw p-value and the Benjamini-Hochberg (BH) adjusted p-value are shown, along with the $\log_2$ fold change (Fontan vs. Control).

Supplementary Table S2: Lipid–Exercise Associations Adjusted for Skeletal Muscle Mass								
**Sl. No**	**Lipid**	**Exercise**	**Beta**	**SE**	**t-statistic**	**p-value**	**Direction**	**Significance**
1	PS 40:5	VE(L/min)	-69.58	21.06	-3.3	0.002	Negative	**
2	PS 40:5	Maximal HR	-55.09	17.63	-3.13	0.003	Negative	**
3	SM 41:2	VE/VCO2	-30.05	8.47	-3.55	0.001	Negative	**
4	Cer 18:0	Peak VO2 (ml/kg/min)	-29.1	8.48	-3.43	0.002	Negative	**
5	PS 40:5	Peak VO2 (ml/kg/min)	-23.54	8.14	-2.89	0.006	Negative	**
6	SM 41:1	VE/VCO2	-15.97	7.42	-2.15	0.038	Negative	*
7	PS 40:5	VCO2	-2.2	0.69	-3.18	0.003	Negative	**
8	PS 40:5	VO2max (L/min)	-1.46	0.53	-2.74	0.009	Negative	**
9	SM 37:2	VO2max (L/min)	1.88	0.52	3.6	0.001	Positive	***
10	SM 37:2	VCO2	2.45	0.7	3.48	0.001	Positive	**
11	SM 42:1	VO2max (L/min)	3.45	1.29	2.67	0.011	Positive	*
12	SM 42:1	VCO2	4.18	1.77	2.37	0.023	Positive	*
13	LPC 20:0	Peak VO2 (ml/kg/min)	26.64	7.13	3.73	0.001	Positive	***
14	SM 37:2	Peak VO2 (ml/kg/min)	28.07	8.14	3.45	0.001	Positive	**
15	LPC(O-22:1)	Peak VO2 (ml/kg/min)	29.23	7.81	3.74	0.001	Positive	***
16	LPC 24:0	Peak VO2 (ml/kg/min)	30.56	8.33	3.67	0.001	Positive	***
17	LPC 18:2	Peak VO2 (ml/kg/min)	32.15	11.63	2.77	0.009	Positive	**
18	LPC 20:1	Peak VO2 (ml/kg/min)	32.92	8.66	3.8	< 0.001	Positive	***
19	LPC(O-20:0)	Peak VO2 (ml/kg/min)	33.86	7.39	4.58	< 0.001	Positive	***
20	LPC	Peak VO2 (ml/kg/min)	52.54	15.98	3.29	0.002	Positive	**
21	LPC(O)	Peak VO2 (ml/kg/min)	56.18	12.9	4.36	< 0.001	Positive	***
22	SM 37:2	VE(L/min)	69.35	22.11	3.14	0.003	Positive	**
23	SM 42:1	VE(L/min)	144.82	52.95	2.73	0.01	Positive	*

Results of linear regression models assessing the association between significantly correlated plasma lipid species and exercise physiology parameters, adjusted for Skeletal Muscle Mass (SMM). Each row represents a lipid–exercise pair that remained statistically significant ($p < 0.05$) in the model: Exercise Parameter ~ Lipid + SMM. Lipid, the lipid species name; Exercise, the corresponding exercise variable; Beta, the regression coefficient; SE, standard error; t-statistic, and associated p-value. ‘Direction’ indicates whether the association is positive or negative, and ‘Significance’ denotes the level of statistical significance using asterisk notation ($p < 0.05$ = *, $p < 0.01$ = **, $p < 0.001$ = ***).
